# Classification Criteria for ANCA Associated Vasculitis – Ready for Prime Time?

**DOI:** 10.1007/s11926-024-01154-9

**Published:** 2024-06-24

**Authors:** Jens Rathmann, Aladdin J. Mohammad

**Affiliations:** 1 Department of Clinical Sciences, Rheumatology, Lund University, Lund, Sweden; 2 Department of Medicine University of Cambridge, Cambridge, UK

**Keywords:** Small vessel vasculitis, Antineutrophil cytoplasmic antibody, Classification, Granulomatosis with polyangiitis, Microscopic polyangiitis

## Abstract

**Purpose of Review:**

This review aims to summarize the evolution and recent developments in the classification of ANCA associated vasculitis (AAV) and to summarize evaluations of the 2022 ACR/EULAR classification criteria of AAV in several cohorts.

**Recent Findings:**

The classification of AAV has been a field of controversy for some time. The parallel existence of classification criteria and disease definitions produced some overlap in classification, leading to challenges when comparing different cohorts. The 2022 ACR/EULAR classification criteria derived from the largest study ever conducted in vasculitis account for significant changes in vasculitis classification with the integration of ANCA and modern imaging. These criteria show good performance compared to previous ones but also raise questions as ANCA serotypes have substantial impact on classification. In addition, there are some discrepancies with earlier agreed histopathological features of AAV disease phenotypes.

**Summary:**

During the last 35 years, several sets of classification criteria have evolved to facilitate epidemiologic studies and clinical trials in AAV. While some of these criteria have been in use for many years, they were criticized due to either not using ANCA or not integrating surrogate markers for vasculitis but also due to overlapping when used in parallel. The long-awaited new ACR/EULAR criteria for AAV were published in 2022 and are the result of a large international study, introducing for the first time ANCA and modern imaging in the classification of AAV. Though the criteria show good performance, they bring several other challenges with practical application.

## Introduction

The systemic vasculitides (SV) comprise several diseases with considerable variation in clinical presentation depending on type and size of blood vessel involved and the organ system affected. Vasculitides are defined as inflammation and necrosis within blood vessel walls with infiltration of inflammatory cells, resulting in blood vessel destruction and impairment of blood supply to the affected territory. Clinical presentations are heterogenous, from single organ involvement to severe multi-systemic, life-threatening disease. The antineutrophil cytoplasmic antibodies (ANCA)-associated vasculitides (AAV) are a subgroup of SV, characterized by vasculitis in small and medium blood vessels. The AAV comprise granulomatosis with polyangiitis (GPA), microscopic polyangiitis (MPA), as well as eosinophilic GPA (EGPA). The three AAV-phenotypes are differentiated according to clinicopathological characteristics defined by the CHCC [1]. ANCA are found in most cases of GPA (mainly Proteinase 3, PR3) and MPA (mainly myeloperoxidase, MPO) but only in 25%- 40% of EGPA (mainly MPO-ANCA). On biopsy, granuloma is a key feature of GPA and EGPA (eosinophil rich) but not MPA [1]. Acute kidney disease and pauci-immune glomerulonephritis are encountered in all the three AAV phenotypes, though much more prevalent in MPA. GPA commonly affects the upper and lower respiratory tract with symptoms from the ENT region as well as primarily nodular lung disease, EGPA can exhibit similar symptoms with a less destructive and more allergic component, but it can also exhibit symptoms as asthma, cardiomyopathy, and polyneuropathy. MPA usually involves the kidney and the lungs, the latter can present as interstitial lung disease even before symptoms of vasculitis in other organ systems. Studies have shown considerable geographic differences in distribution with MPO-positivity and MPA being more frequent in Asia, and PR3-positivity and GPA more frequent in western countries [2]. AAV are severe diseases that can carry high risk of mortality if untreated [3]. The prognosis of AAV has greatly improved since the introduction of glucocorticosteroids (GCs) and cytotoxic/ immunosuppressives in the treatment arsenal in 1970s [4]. The rarity of AAV poses a challenge to epidemiologic and clinical studies due to small number of cases and paucity of large well-validated cohorts [5]. An important prerequisite when studying these rare and heterogenous diseases is a common nomenclature of agreed definitions and classification criteria. These are essential to compare results from different regions, cohorts, and time periods. Unlike diagnostic criteria that aim to differentiate vasculitis from other diseases, classification criteria aim to differentiate one type of vasculitis from another. Classification can be based on different parameters. Histological parameters have been widely used, describing the size of affected vessels or the type of inflammatory process (granulomatous, necrotizing, eosinophilic etc.). Other parameters are mechanisms for example secondary to infectious agents, formation of antibodies or immune-complexes, further organ tropism or localized versus systemic involvement and classification according to clinical presentation.

Since the earliest proposed classification of SV in the middle of the last century, several key efforts have been made in the field of classification and definition, thereby greatly advancing the research on vasculitis with clinical trials and epidemiological studies.

During the last 35 years, the American College of Rheumatology (ACR) classification criteria and the Chapel Hill Consensus Conference (CHCC) definitions have dominated in the field of epidemiologic studies of SV and facilitated these kinds of studies. Since their introduction, advancements in immunology and imaging have revolutionized our understanding of the pathogenesis and epidemiology of SV. Further, the availability of better treatment options, both in induction of remission and maintenance, has transformed the traditional perception of SV from a fatal disease to one characterized by relapses and remissions, allowing more individuals to live with these conditions. These developments have prompted a re-evaluation of the necessity for new criteria, considering the recent advances in our understanding of SV.

Beginning in 2010, a large international collaborative study to develop new classification and diagnostic criteria (DCVAS) has been conducted [6]. This study has yielded classification criteria for the AAV, Takayasu arteritis (TAK) and giant cell Arteritis (GCA).

In this review, we aim to trace the evolution of the classification of AAV from its inception, beginning with a brief historical overview, through the development of the most widely utilized criteria over the past 30 years and finally a discussion of our evaluation as well as results of other researchers when using the new ACR/EULAR (European Alliance for Associations for Rheumatology) criteria.

## Epidemiology

Epidemiologic studies demonstrated variable incidence and prevalence of AAV across different geographic areas in the world [7]. Reasons for these variations are probably related to differences in methodology used in case retrieval, case definition and classification. Furthermore, differences in geographic and genetic background as well as different time periods. Several earlier studies have indicated rising incidences of the AAV during the last 30 years [8–10]. Most recently, a 23- years incidence study on epidemiology of AAV by our group found an incidence of 30.1/million, comparable incidence of GPA (15.4/million) and MPA (12.8/million), EGPA (1.8/million) incidence is considerably lower [11]. The rising incidence reported by previous studies, could not be verified in our area of southern Sweden in our large population-based cohort, with cases from 23-years and use of same retrieval method and classification criteria overtime. We believe that the apparent increase in incidence of AAV reported by previous studies was mainly impacted by the introduction of ANCA testing in mid 1980s [12], the ACR 1990 classification criteria in 1990 [13•], as well as the CHCC definitions in 1994 [14•].

### Historical Overview of Classification Criteria

Early descriptions of symptoms compatible with vasculitis can be found in ancient medical literature, for example Hippokrates description of a case with oral and genital ulcers and ocular inflammation [15], symptoms today included in current criteria for Behçet’s disease [16]. The description by Kussmaul and Maier [17] of a patient with fever, weakness, weight loss and pain, where autopsy later showed nodular thickening of medium sized arteries is often cited as the first modern description of vasculitis. In this description from 1866, the term periarteritis nodosa was first introduced, a term, along with its alternative polyarteritis nodosa (PAN), used synonymous for all vasculitis for many years. Different vasculitis syndromes were often described in relation to their similarity or diversity from PAN. Klinger described a rhinogenic granulomatosis [18] later known as Wegener´s granulomatosis [19], Churg and Strauss described a syndrome including vasculitis, asthma and eosinophilia, later bearing their names [20]. In the twentieth century, efforts to better define and classify vasculitis were first made by the pathologist Pearl Zeek, who combined literature review and own observations to suggest 5 different forms of necrotizing vasculitis in 1953. Zeek introduced the concept of vessel size, later refined by Giliam and Smiley [21], however Wegener´s granulomatosis or Takayasu Arteritis were not included in Zeek´s classification, presumably as they had not been described in English literature at that time. In 1990 the ACR developed a set of classification criteria for seven forms of systemic vasculitis. The ACR 1990 criteria did not include classification criteria for MPA, neither used ANCA serology. To address these and further questions a consensus meeting was held in Chapel Hill, USA 1992. The results, definitions of different SV published in 1994 [14•] and revised in 2012 [1], were widely used in epidemiologic studies. As the parallel use of classification criteria and definitions produced overlap and sometimes confusion, a group of vasculitis experts introduced an algorithm incorporating both systems, the European Medicines agency (EMA) algorithm in 2007. In the last 2 years classification criteria endorsed by ACR and EULAR have been published for the AAV as well TAK and GCA, these criteria were the result of the largest study ever conducted in vasculitis the DCVAS [6].

### Classification According to ANCA Serotype

ANCA were first observed by Davies et al. in 1982, patients with segmental glomerulonephritis exhibited a factor in their sera that stained the cytoplasm of neutrophil granulocytes [22]. A few years later van der Woude et al. [12] described a cytoplasmic immunofluorescence pattern of antibodies directed against components in granulocytes in patients with active GPA (then designated as ACPA (anti-cytoplasmic antibodies), suggesting an association between these antibodies and vasculitis. During the following years PR3- ANCA [23] and MPO-ANCA [24] were identified as target antigens for the cytoplasmic and perinuclear pattern respectively. ANCA are routinely used in clinical context today. Due to variability of IIF and good performance and fast evolution of immune assays, latest consensus now recommends the use of ELISA as first line test for ANCA [25]. There is an ongoing discussion among researchers in the field, regarding the use of ANCA serotypes as a classification system for these diseases [26–28]. The discovery of distinct genetic subsets of GPA and MPA that exhibit stronger association with serotype than phenotype [29], a finding that even could be made for EGPA when stratifying according to MPO-status [30] supports a shift towards serotype classification. As we try to demonstrate below, it can be argued that this shift clearly is represented with respect to the considerable weight granted to ANCA in the new ACR-EULAR criteria.

## Currently Used Criteria and Definitions

### American College of Rheumatology Criteria 1990 (ACR 1990)

The ACR criteria from 1990 for SV were the first to combine different parameters into one set of classification criteria. The criteria were developed from a dataset with 1020 cases of vasculitis from North America (USA, Canada, and Mexico). Through identification of features typical for different types of vasculitis, criteria for seven different forms of systemic vasculitis (GPA, EGPA, GCA, TAK, IgA vasculitis, PAN and hypersensitivity vasculitis) [13•] were proposed. The criteria were widely used and facilitated clinical and epidemiological research in the field of vasculitis, however with time several shortcomings became obvious: i) the criteria lacked ANCA, though assays became widely available in the following years, ii) MPA was not included as a separate disease entity, iii) the underlying dataset only included cases from North America not accounting for geographical differences in phenotype distribution, in addition certain subtypes were more represented than others in the dataset, and iv) cases could be classified to different subtypes at the same time. Though not intended as such, the criteria were used for diagnosis in individual cases in routine clinical practice. The criteria performed poorly in an evaluation as diagnostic criteria [31]. The criteria had considerable impact on epidemiologic research in vasculitis.

### Chapel Hill Consensus Conference (CHCC)

In 1992 a meeting of a group of experts in Chapel Hill yielded a consensus document [14•] defining different types of vasculitis and establishing a nomenclature. The experts emphasized that the goal of the CHCC was not to develop classification or diagnostic criteria rather give definitions and define a common nomenclature. Ten different types of vasculitides were defined according to histological and clinical criteria and grouped on predominantly affected vessel size. The system accounted for the presence of ANCAs. The meeting further established MPA as a disease entity separate from GPA and EGPA as well as the association of ANCA with these small vessel vasculitides. As cases earlier attributed to PAN now became MPA, the former became a very rare disease. In an update 2012 [1], the group of small vessel vasculitides was further divided into the immune-complex related- or pauci-immune ANCA-associated vasculitides. Regarding the AAV, the consensus suggested using a prefix indicating ANCA reactivity, the concept of surrogate criterion (cavitary lung lesion on imaging can be a surrogate criterion for granulomatous disease without available histologic examination) is discussed (as it was in 1994) in the publication but without detail or specific guidance. In 2000 a study by Sorensen et al. tried using the CHCC definitions with surrogate markers as diagnostic classification criteria but it was not successful [32]

### European Medicines Agency (EMA) Algorithm 2007

The two available widely used systems, the ACR 1990 and the CHCC 1994 definitions performed differently when applied in parallel in same cohort [33], leading to overlapping as patients may be classified into different disease phenotypes or remain unclassified by one of these systems. To avoid this overlapping and to gain a consensus on how the ACR 1990 classification and CHCC 1994 definitions should be used in epidemiologic studies, a group of experts met in London in 2004 and agreed on a stepwise algorithm [34•]. The algorithm, which is widely known as Watts algorithm or EMA algorithm, integrated ACR 1990 and CHCC in a hierarchic way. The algorithm starts with ACR EGPA [35] as it had the highest sensitivity and specificity of the ACR 1990 criteria, then the following steps ACR 1990 GPA [36] and CHCC 1994 MPA are applied. The algorithm also introduced the combined use of ANCA and surrogate markers of vasculitis and/or granuloma. The EMA algorithm has been used in several epidemiological studies in the last 15 years [8, 10, 11] and showed minimum of overlapping or unclassified cases.

### The Diagnostic and Classification Criteria in Vasculitis (DCVAS)

The evolution of imaging, widespread use of ANCA-testing and the improved knowledge and understanding of pathophysiology in vasculitis initiated a discussion on revision of classification of the primary vasculitides. The multinational observational DCVAS study was announced in 2010 [6] with the goal to develop and validate diagnostic criteria and improve and validate classification criteria. The project accumulated an impressive cohort and invested great efforts and applied advanced statistics to develop totally new criteria that are intended to be used independent of the prior ones. However, succeeding with the endeavour to even develop diagnostic criteria seems highly unlikely at this point [37]. The DCVAS final cohort included 6991 patients from 136 sites in 32 countries (Europe 59%, North America 21%, other regions 20%). Patients ≥ 18 years with a diagnosis within 2 years of GPA, EGPA, MPA, GCA, anti glomerular basement membrane disease (anti GBM), PACNS, IgA vasculitis, aortitis, other large vessel vasculitis, PAN (within 5 years) or TAK (within 5 years) or a condition mimicking vasculitis secondary to tumour, infection, or other inflammatory condition as diagnosed by the submitting physician, were included. First a final set of high standard cases for each subtype was identified and included into the study, in the next step a set of candidate items were identified and further refined by expert opinion and a data driven approach (exclusion of items with low prevalence and/or not clinically relevant). In the next step a regression model was used which yielded the final criteria that were in the next step validated in a validation data set. Comparators used in the derivation and validation of the criteria for each subtype were the other AAV or to a minor extent other small or medium vessel and not large vessel vasculitides with generally very different phenotype. All these efforts have culminated in the introduction of new classification criteria for the AAV [38••, 39••, 40••] in 2022 as well as for GCA [41] and TAK [42].

### The 2022 ACR/EULAR 2022 Criteria for AAV

The new 2022 ACR/EULAR (in the following text designated as ACR/EULAR) criteria derived from the largest cohort ever established in systemic vasculitis. The cohort included mainly patients from western countries, however the recruited cases were much more international than those used for earlier criteria as the ACR1990. Unlike the ACR1990 criteria diagnosis of the submitting physician was reviewed by an expert panel, thereby minimizing investigator bias. The criteria account for the evolution of diagnostics and clinical practice by including ANCA and modern radiology. Another innovation is the weighting of items to reflect clinical importance of features.

The final criteria include a different number of weighted items for the subtypes with threshold scores to be classified. Some items can have negative impact on the score, as for example nasal symptoms with MPA, or the presence of eosinophilia with GPA and MPA.

The following is a short summary of main items in respective criteria, described in detail in Table 1:

**Table 1:** The 2022 ACR/EULAR classification for AAV criteria, modified from original publications [38••, 39••, 40••], prerequisite for classification is the diagnosis of small-medium vessel vasculitis established and vasculitis mimics excluded

	GPA		MPA		EGPA	
Clinical	**Nasal** *Crusts, discharge, ulcers, congestion, perforation*	** + 3**	**Nasal** *Crusts, discharge, ulcers, congestion, perforation*	**-3**	**Asthma**	** + 3**
	**Cartilage** *Ear, nose, stridor, endobronchial, saddle nose*	** + 2**			**Nasal Polyps**	** + 3**
	**Hearing loss**	** + 1**			**Mononeuritis multiplex**	** + 1**
Lab, imaging, serology	**Positive cANCA or PR3**	** + 5**	**Positive pANCA or MPO**	** + 6**	**Blood eosinophils ≥ 1 × 10**^**9**^**/L**	** + 5**
	**Chest imaging** *Nodules, mass, cavitation*	** + 2**	**Chest imaging** *Fibrosis/ILD present*	** + 3**	**Extravascular eosinophil rich inflamation on biopsy**	** + 2**
	**Granuloma on biopsy**	** + 2**	**Pauci-immune GN on biopsy**	** + 3**	**Positive cANCA or PR3**	**-3**
	**Sinus imaging** *Effusion, consolidation*	** + 1**	**Positive cANCA or PR3**	**-1**	**Hematuria**	**-1**
	**Pauci-immune GN on biopsy**	** + 1**	**Blood eosinophils ≥ 1 × 10**^**9**^**/L**	**-4**		
	**Positive pANCA or MPO**	**-1**				
	**Blood eosinophils ≥ 1 × 10**^**9**^**/L**	**-4**				
Scoring	Sum scores 10 items ≥ 5 = GPA		Sum scores 6 items ≥ 5 = MPA		Sum scores 7 items ≥ 6 = EGPA	

*cANCA*, cytoplasmic antineutrophil cytoplasmic antibody, *pANCA,* perinuclear ANCA, *PR3* proteinase-3. *MPO*, myeloperoxidas, *GN,* Glomerulonephritis, *ILD*, interstitial lung disease

#### GPA

The GPA dataset consisted of 1537 cases (724 GPA and 813 comparators), 20% were used in the validation set. The final criteria consist out of 10 weighted items with a threshold score of 5 needed for classification. The authors report a sensitivity of 92.5% and specificity of 93.8% in the validation data set [39••].

#### MPA

The MPA dataset consisted of 1113 cases (291 MPA and 822 comparators), 50% were used in the validation set. The final criteria consist of 6 weighted items with a threshold score of 5 needed for classification. A sensitivity of 90.8% and specificity of 94.2% in the validation set is reported [40••].

#### EGPA

The EGPA dataset consisted of 1113 cases (226 EGPA and 887 comparators), 50% were used in the validation set. The final criteria consist of 7 weighted items with a threshold score of 6 needed for classification. The authors report 84.9% sensitivity and 99.1% specificity in the validation set [38••].

### Evaluation of the ACR/EULAR Criteria

An important question with new classification criteria or disease definition is if they improve shortcomings of earlier criteria and if they exhibit better sensitivity and specificity. The 1990 ACR-criteria did not include MPA, but this was added with the CHCC as well as ANCA. The lack of guidance concerning ANCA as a surrogate marker could be improved by the EMA algorithm. ACR/EULAR introduces weighted items with positive and negative scores. In addition, ANCAs are introduced, with considerable weight. Further, new items such as interstitial lung disease (ILD) are introduced. ILD has been observed especially in patients with MPO-associated disease [43], often before the onset of vasculitis [44] and has therefore become more recognized in AAV.

As of January 2024, the new criteria have been evaluated in studies from South Korea, Turkey, Netherlands, Japan, Portugal, India, Ukraine, and Sweden (Table 2). Some reports are only available as conference abstracts. Below is a summary of main findings of some of these evaluations:

**Table 2:** Characteristics of selected cohorts classified by EMA and 2022 ACR/EULAR and agreement between the systems (if provided)

Country (Refernce)	South Korea GPA [46]	South Korea MPA [47]	South Korea EGPA [45]	Turkey [49]	Japan [50]	Sweden [54]
No of Cases	65	117	51	164	477	374
Age, mean (± SD)/median (IQR), years	61 ± 20	64 ± 18	54 ± 22	49.6 ± 14.4	73****	67.5 (55—77)
Sex, female %	60	63	69	41	57	47
MPO positive, *n* (%)	28 (43) *	114 (97) *	25 (55) *	116 (71)	404 (85)	161 (43)
PR3 positive, *n* (%)	36 (54) *	4 (3) *	5 (11) *	37 (22)	42 (9)	188 (50)
ANCA neg, *n* (%)	5 (3)	3 (2)	24 (53)	11 (7)	31 (6)	25 (7)
EMA classification, number of cases						
GPA, *n* (%)	65 (100)			121 (74)	86 (18)	192 (51)
MPA, *n* (%)		117 (100)		21 (13)	276 (58)	159 (43)
EGPA, *n* (%)			51 (100)	8 (5)	42 (9)	23 (6)
Unclassified, *n* (%)				14 (8)	73* (15)	0
ACR/EULAR classification, number of cases						
GPA, *n* (%)	48 (74)	3 (3)	1 (2)	117 (71)	47 (10)	199 (53)
MPA, *n* (%)	16 (25)	113 (96)	3 (6)	24 (15)	361 (75)	136 (36)
EGPA, *n* (%)	0	0	44 (86)	10 (6)	51 (11)	22 (6)
Unclassified, *n* (%)	1 (1)	1 (1)	3 (6)	11 (7)	29 (6)	13 (3)
Double classified, *n* (%)	0	3 (3)	7 (13)	2 (1)	11 (2) ***	4 (1)
Granuloma classified to MPA, *n* (%)	4 (6)	0	0	4 (3)	4 (1)	4 (1)
ILD in MPA item ACR/EULAR, *n* (% of total MPA)	8 (50)	58 (50)	na	3 (12)	176 (49)	15 (11)
EMA GPA to ACR/EULARMPA, *n* (%)	16 (25)	na	na	4	35 (7)	12 (3)
EMA MPA to ACR/EULARGPA, *n* (%)	na	3 (3)	na	Not provided	15 (3)	35 (9)
Type of cohort	Single center	Single center	Single center	Hospital Records two academic centers, Ankara	Nationwide inception cohort from Remission study	VR (population-based)
Type of classification	EMA,ACR/ EULAR	EMA,ACR/ EULAR	EMA,ACR/ EULAR	ACR1990, EMA, ACR/EULAR	EMA,ACR/ EULAR	EMA, ACR/ EULAR, Serotype
Agreement EMA and ACR/EULAR						
GPA, %	74	na	na	91.3	Not provided	85
MPA, %	na	97	na	90.5	Not provided	75
EGPA, %	na	na	86	100	Not provided	96

*Frequencies as reported in the publications, total percentage may exceed 100% due to double positivity, **71/73 cases could be classified to a single category with ACR/EULAR, ***Total percentage exceeds 100% due to double classified included, ****Approximation, as median is only provided for subtypes in publication. *GPA*: Granulomatosis with polyangiitis, *MPA*: Microscopic polyangiitis, *EGPA*: Eosinophilic GPA, *ILD*: Interstitial lung disease, *EMA*: European Medicines Agency, *VR*: Vasculitis register

### South Korea

Three separate studies for the AAV-subtypes from Korea have investigated reclassification of AAV-cohorts into disease phenotypes using the ACR/EULAR. For EGPA [45] the concordance rate with EMA was 86.3%, 7 former EGPA cases met criteria for two subtypes with the new criteria and 3 cases could not be classified. The authors consider the exclusion of the fixed pulmonary infiltrate item from ACR1990 as reason why these 7 EGPA cases could not be classified the same way with ACR/EULAR. Concerning GPA only 74% of cases remained GPA with ACR/EULAR [46], most were reclassified as MPA, predominantly due to MPO-ANCA positivity. The authors also encountered cases with granuloma on biopsy, now being classified as MPA and suggest score modification of the granuloma item and other clear GPA surrogates in the new criteria. In a further study on MPA cases [47] classified using the EMA algorithm, a high rate of agreement was observed with the ACR/EULAR (96.6%). A small number of patients were classified to GPA and MPA due to positivity to both antibodies. Almost 50% of the MPA cohort exhibited ILD, a feature now having considerable weight towards MPA classification. However, the authors commented that the definition of ILD is broad and a consensus on when this item should be scored is needed. In summary the authors criticize the high score assigned to MPO-ANCA and the lack of consideration of MPA-specific histopathological findings in the new criteria.

### Netherlands

Hospital records of 337 patients with AAV from a tertiary centre with a confirmed clinical diagnosis were used in this evaluation [48]. After exclusion of a relatively high number of cases due to unclear diagnosis or insufficient records, 264 (GPA = 183, MPA = 54, EGPA = 27) patients were reclassified with ACR/EULAR. Information on prior classification with established criteria is not provided. The study reports sensitivities of 88% for GPA, 94% for MPA and 74% for EGPA, 17 patients could not be classified by ACR/EULAR. ANCA-serology is provided for 261 cases with 59% PR3-positives, 31% MPO-positives and 9% ANCA-negatives. The researchers noted a large impact of serology on reclassification to GPA and MPA reflected in the finding that 94% of GPA patients were PR3 positive and 100% of the MPA patients MPO-positive in their results. The study therefore considers the new criteria to be step closer to a serology-based classification.

### Turkey

A Turkish study [49] with 164 patients with AAV (clinical diagnosis: 77% GPA, 15% MPA, 8% EGPA) diagnosed between 2016 and 2022, recruited from two academic centres and reclassified by ACR-EULAR, describes a kappa for agreement with EMA classification of 0.79 for GPA, 0.7 for MPA and 0.82 for EGPA. There were two double-classified cases (MPA + GPA) and four former GPA patients are now classified to MPA due to positive anti-MPO. Only 7% of cases could not be classified (2 with granuloma on biopsy). Four patients, earlier classified as GPA, all with granuloma on biopsy, were now assigned MPA due to MPO positivity.

### Japan

Two nationwide inception cohorts were used in this evaluation study [50]. All patients were classified using EMA algorithm and were then validated using the ACR/EULAR 2022 criteria. A total of 477 patients were included in the study. The majority of the cases were MPO-ANCA positive (85%), 9% PR3-ANCA positive and 6% were ANCA-negative. Using these two classifications, MPA was the dominant phenotype (EMA:57%, ACR/EULAR: 76%). Details on the composition of the cohort are given in Table 2. A high proportion of patients being either classified to EGPA or MPA by EMA retained this diagnosis with ACR/EULAR, in contrast, 41% of EMA GPA patients were instead classified to MPA. 82.1% of ACR/EULAR GPA patients were PR3-positive whereas 100% of the ACR/EULAR MPA patients exhibited MPO-positivity, reinforcing the weight of serology in ACR/EULAR. In this study, a small number (*n* = 4) of patients classified to MPA with ACR/EULAR exhibit granuloma on biopsy. A large proportion of patients that could not be classified by EMA, could be classified by ACR/EULAR, all of these patients were MPO-positive and a majority exhibited ILD, thus most were assigned to MPA. The new criteria might be more useful in Asian cohorts with dominating MPO-positivity and a high degree of ILD. On the other hand the authors also report difficulties in classifying ILD in MPO-positive cases, ILD has been shown to occur in EGPA and GPA in a Japanese cohort study but most cases will be classified to MPA with ACR/EULAR.

### Other Evaluations

A Portuguese study, presented as meeting abstract including 152 patients with MPA and GPA also compared EMA and ACR/EULAR [51], reporting a shift in classification of 34.7% earlier EMA GPA-classified patients to either MPA or unclassifiable vasculitis. Even in this cohort there were a few patients with EMA GPA and granuloma on biopsy now classified to MPA using the new ACR/EULAR criteria. An Indian evaluation, presented as abstract [52] with patients from the Indian vasculitis registry, reports on 302 relatively young AAV patients with a mean age of 42.9 ± 14.7, classified by EMA, 2022 ACR/EULAR and ACR1990. Classification was then compared to clinicians diagnosis, considered gold standard. The new criteria demonstrated highest agreement in all categories. In ANCA negatives, predominant ocular or CNS GPA were missed by the new criteria. An Ukrainian study, presented as abstract reports better performance than ACR1990 and EMA in 42 patients with AAV, but 20% of patients can either not be classified or are assigned two diagnoses [53].

### Sweden

**Fig. 1:**
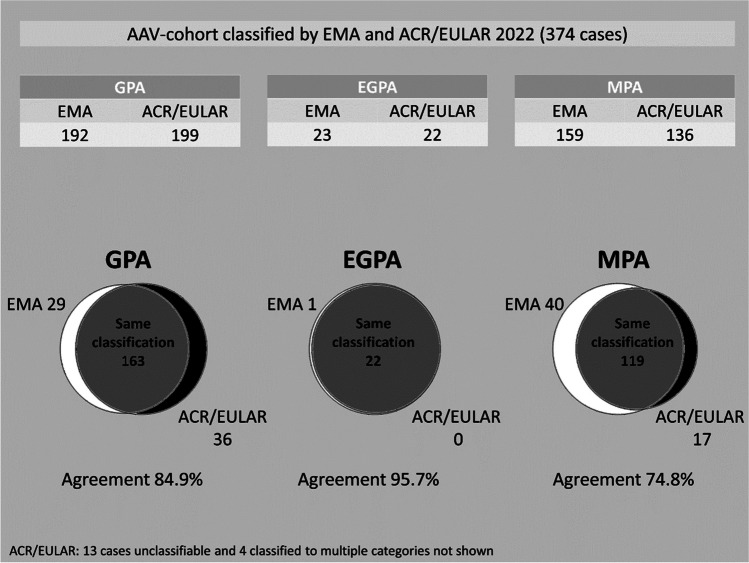
Classification outcome in Skåne-AAV cohort with permission from Rheumatology (Oxford) [54]

Our evaluation of the new criteria employed a population-based cohort of 374 (47% female) validated cases with AAV [54], previously classified by the EMA algorithm. We compared classification outcome of ACR/EULAR to i) EMA and to a ii) strictly ANCA serology-based classification without any impact from clinical or histologic features. In general, the new ACR/EULAR criteria showed good agreement with EMA, with 96% for EGPA, 85% for GPA and 75% for MPA. We observed a low number of cases that could not be classified (3.5%) or are classified to two categories (1.1%). Fourteen percent of cases (69% MPA, 31%GPA) are assigned a different diagnosis with the new criteria due to ANCA-specificity. We further observed 4 cases exhibiting granuloma on biopsy but assigned to MPA. Regarding ANCA serology-based classification; very high agreement was observed with 98.9% for GPA and 84% for MPA, upon excluding cases with EGPA (*n* = 23), these numbers increased to 99.5% and 88.2% respectively (Fig. 1). There were 13 unclassifiable cases, 6 were ANCA-negative which corresponds to a third of all ANCA-negatives in the cohort. This might indicate difficulties classifying ANCA-negatives with ACR/EULAR. ANCAs are less frequent in EGPA, the predominant ANCA serotype MPO, presumably favouring classification to EGPA in certain cases is not even included in the EGPA category, the other serotype is included with negative three points thus making classification to EGPA more unlikely. Of 23 EGPA (EMA) in the Swedish cohort eight are MPO positive which does not impact classification at all, there are no PR3 positive patients, but assuming all the EGPA patients were indeed PR3 positive, all would qualify to be classified to EGPA as they all generate sufficient score with the other items. Of course, some of these patients would then even be classified as GPA, so many would shift to the double classified group. This means inclusion of ANCA in the EGPA category does not change the classification towards EGPA score in our patients at all.

### Challenges in Using the new ACR/EULAR 2022 Criteria

Based on available evaluations, there are some challenges when applying/using the new 2022 ACR/EULAR classification criteria of AAV; these are summarized as:

1. Overlapping classification; cases are classified to more than one phenotype.
2. Unclassifiable cases are described in all studies.
3. Disagreement with current understanding of AAV histopathology; the CHCC defined granuloma as a key feature of GPA, but cases with granuloma on biopsy are classified to MPA in several of the cited studies. Granuloma generates low score in the GPA towards GPA classification, but it is not included in the MPA at all, thus granuloma can now be a feature of MPA given MPO positivity or other items classifying towards MPA are present. In this case a serotype feature (MPO-positivity) clearly outweighs a phenotypic feature as the weight of granuloma is low or not existing in the MPA category.
4. Interstitial lung disease (ILD) is poorly defined in the ACR/EULAR criteria. It is not clear when the criteria of ILD are met. However, given the results from the Japanese study, the addition of ILD is important, as more patients can be classified in populations where ILD as a clinical manifestation of AAV is common.
5. Challenges related to ANCA
  - ANCA-negative cases: potentially ANCA-negative cases could be become more difficult to classify, which our own findings support, however more studies are needed in this subgroup for clarification.
  - ANCA weight and test method: the inclusion of immunofluorescence in the criteria can, in our eyes, be problematic, as positive IIF is observed in several other diseases, we consider the use of ELISA based test to be more specific.
  - A considerable number of patients are classified to another phenotype. This applies primarily to MPO-positive patients who will often be assigned MPA primarily due to MPO positivity. Features typical for non MPA phenotypes might, due to ANCAs weight, be “outscored” and classified to MPA.

## Conclusion

There have been efforts to classify vasculitides for many decades. Considerable progress has been made overtime facilitating epidemiological and clinical research in the field. Classification criteria from different time-periods reflect the knowledge on a given disease at that time and evolution of diagnostic possibilities need to be reflected in newer criteria. The ACR1990 criteria were a milestone but lacked ANCA, as they were developed before the widespread introduction of ANCA. Later efforts as CHCC and EMA tried to improve shortcomings but raised new question and challenges. So new criteria might improve deficits of the older ones by incorporating new knowledge and diagnostic possibilities, at the same time new questions arise. This pattern seems to continue with the 2022 ACR/EULAR criteria, on the one hand the criteria incorporate ANCA and new imaging and they were developed with advanced statistical methods and the largest cohort ever established in AAV. They are easy to use with an innovative scoring system and they show good performance compared to the prior systems. On the other hand, unclassifiable cases remain, and questions as the disagreement with earlier definitions (granuloma) arise. The criteria grant considerable weight to ANCA, indicating a clear shift towards serotype classification. As we could show the differences compared to a pure serotype classification (in GPA and MPA) are quite low. The next step could be a shift to serotype classification, as others have argued [55]. Recent discoveries including big data cluster analysis however can be interpreted as an argument to not abandon phenotypic characteristics totally [55].
